# Antimicrobial resistance, virulence genes, and phylogenetic characteristics of pathogenic *Escherichia coli* isolated from patients and swine suffering from diarrhea

**DOI:** 10.1186/s12866-022-02604-z

**Published:** 2022-08-16

**Authors:** Kyung-Hyo Do, Kwangwon Seo, Wan-Kyu Lee

**Affiliations:** Veterinary Medical Center and College of Veterinary Medicine, Chungbuk National University, Cheongju, 28644 Republic of Korea

**Keywords:** Swine, Patients, Diarrhea, *Escherichia coli*, Virulence factors, Antimicrobial resistance, Multi-locus sequence typing

## Abstract

**Background:**

*Escherichia* (*E.*) *coli* causes colibacillosis in swine and humans, and is frequently associated with antimicrobial resistance. In this study we aimed to compare antimicrobial resistance, O-serogroups, virulence genes, and multi-locus sequence type of *E. coli* between isolates from pigs and patients suffering from diarrhea, and the most prevalent pathogenic *E. coli* strain from swine isolates in Korea.

**Methods:**

We tested 64 and 50 *E. coli* strains from pigs and patients suffering from diarrhea for antimicrobial susceptibility test, virulence genes, O-serogroups, and multi-locus sequence typing.

**Results:**

We confirmed that isolates from swine showed significantly higher resistance than from those from patients, especially to fluoroquinolone (ciprofloxacin: 37.5 and 10.0%; norfloxacin: 29.7 and 8.0%, respectively). Stx1 (46.0%) was most frequently detected in patients followed by stx2 (38.0%). There was no significant difference in stx2 (swine: 23.4%, patients: 38.0%). In isolates from patients, O157 (12.0%) was the most prevalent O-serogroup, and two isolates (3.1%) from pigs were confirmed to have O157. Additionally, sequence type (ST) 10 (swine: 6 isolates, patients: 2 isolates) and ST 88 (swine: 2 isolates, patients: 1 isolate) were simultaneously detected.

**Conclusions:**

We found that both isolates from swine and human had the stx2 gene, which could cause severe disease. Moreover, antimicrobial resistance was significantly higher in pigs than in patients. These results suggest that pig could act as a reservoir in human infection and antimicrobial resistance could be transferred to human from pigs.

**Supplementary Information:**

The online version contains supplementary material available at 10.1186/s12866-022-02604-z.

## Background

*Escherichia coli* (*E. coli)* causes colibacillosis, a common disease that occurs in pigs and humans [1]. Colibacillosis in pigs has a significant economic impact on the pig farming due to its association with high rates of morbidity and mortality [2]. Moreover, pathogenic *E. coli* causes diarrhea and hemorrhagic colitis in humans, with life-threatening complications, such as hemolytic uremic syndrome [3].

Antimicrobial agents are frequently used for the treatment of colibacillosis [4]. In Korea, the largest number of antimicrobials was sold for use in pigs (55%, 507 tons), which was higher than that sold for use in poultry (17%, 155 tons) and cattle (11%, 99 tons) [5]. Consequently, antimicrobial resistance was much higher in swine isolates than that in isolates of other livestock [5]. Since antimicrobial resistance can be transferred from pigs to humans, there is a need for the surveillance of antimicrobial resistance in pigs [6].

The pathogenicity of *E. coli* is determined by virulence genes (toxin and adhesin) and/or O-serogroups [7]. The frequency of these virulence factors is known to vary over time and based on the host. Although a variety of O-serogroups have been associated with colibacillosis, a limited number of serogroups have been reported for specific disease, such as postweaning diarrhea, edema disease, and hemorrhagic colitis [8, 9]. Pigs are considered the primary reservoirs of pathogenic *E. coli* which can lead the contamination of food products and human infection [2]. Therefore, it is important to establish the clonal relationship between strains from different hosts and diseases to assess the risk of zoonotic infections [10].

Although there have been many studies that focused on the antimicrobial resistance of *E. coli*, but most studies analyzed the antimicrobial resistance of commensal *E. coli*. There have been fewer studies on the correlation between antimicrobial resistance and virulence factors of *E. coli* in diarrheic pigs and in patients suffering from diarrhea. In this study, we aimed to compare antimicrobial resistance, O-serogroups, virulence genes, and multi-locus sequence type (MLST) of most dominant pathogenic *E. coli* from pigs with the isolates obtained from patients suffering from diarrhea in Korea.

## Methods

### *E. coli* strains

For this study, the most dominant 64 pathogenic *E. coli* strain from piglets suffering from edema disease and postweaning diarrhea in Korea between 2008 and 2016 were used, according to a previous study [9]. There were 64 different pig herds (50 to 100 sows per herd) on the studied farms. Twenty-nine strains of enterotoxigenic *E. coli* (ETEC), 28 strains of shiga toxin-producing *E. coli* (STEC), and seven strains of ETEC/STEC from pigs were selected. From 50 different patients suffering from diarrhea, 50 *E. coli* strains were isolated between 1981 and 2014, and kindly provided by the National Culture Collection for Pathogens (NCCP), Korea. Detail information for strains in this study were described in Table S1. The isolates were classified as each pathotype according to following criteria (2, 3, 8, 9): ETEC (encoding LT, STa, STb, EAST-1, or any combination thereof), STEC (encoding stx1, stx2, stx2e, or any combination thereof), EPEC (encoding eae), EAEC (encoding aggR), and EIEC (encoding ipaH). For isolating these strains, aseptically collected intestinal and swabbed fecal samples were inoculated onto MacConkey agar (Becton Dickinson, MD, USA). After overnight incubation at 37 °C, only pure cultured colonies that were pink in color were selected and transferred to blood agar (Asan Pharmaceutical, Korea). Suspected colonies were identified as *E. coli* by using the VITEK II system (bioMéreiux, Marcy I’Etoile, France). The tested isolates were stored in 50% glycerol stock at − 70 °C until further characterization.

### Detection of virulence gene and O-serogroups

Reference *E. coli* strains provided by the animal and plant quarantine agency (Korea) were used as positive controls for polymerase chain reaction (PCR) analysis, they included: 7805 (F4:LT:STa:STb:EAST-I:paa), 6611 (stx1:stx2:eae: EAST-I:paa), 1033 (F18:AIDA-I), 2316 (F6:STa:STb:EAST-I:paa), and 13,316 (F5:F41:STa:paa); and 3463 was used as a negative control. Template DNA for PCR analysis was extracted using the boiling method. PCR test described below was conducted for analyzing F4, F5, F6, F18, F41, eae, paa, AIDA-I, stx1, stx2, aggR, ipaH, LT, ST, and EAST-I as previously described [9].

The reaction volume (20 μL) was composed of 2 x EmeraldAmp Master Mix (TaKaRa, Japan), 2 μM of each primer, and 3 μL of DNA template. TaKaRa PCR Thermal Cycler Dice Gradient TP600 (TaKaRa, Japan) was used for performing the PCR analysis. After amplification, the resultant products underwent electrophoresis on 2% agarose gel using Mupid-exu AD140 (TaKaRa, Japan), stained with BlueMango (BioD, Korea), and was confirmed using BluePad (BioD, Korea). O-serogroup typing was performed using the slide agglutination technique in the Animal and Plant Quarantine Agency (Korea) using rabbit antisera purchased from Serum Staten Institute (Denmark).

### Antimicrobial susceptibility test

The following 21 antimicrobial agents were selected by referring to the Clinical and Laboratory Standards Institute (CLSI) guidelines and were used in this study: gentamicin; streptomycin; neomycin, kanamycin, amikacin, amoxicillin / clavulanate, cephalothin, cefazolin, cefoxitin, cefepime, nalidixic acid, ciprofloxacin, norfloxacin, tetracycline, doxycycline, ampicillin, trimethoprim / sulfamethoxazole, chloramphenicol, colistin, and clindamycin [11]. Each antimicrobial disc was purchased from Becton-Dickinson (BD, USA). Antimicrobial susceptibility testing was carried out using the Kirby Bauer disk diffusion method [12]. The CLSI standards *Enterobacteriaceae* breakpoints were used for the interpretation of resistance [11]. Strains resistant to three or more CLSI subclass drugs according to the Magiorakos criteria were considered as multidrug resistant strains [13].

### Multi-locus sequence typing (MLST)

All processes, including genomic DNA extraction, PCR amplification, Sanger sequencing, and assembly were performed by Macrogen (Macrogen, South Korea). Genomic DNA was extracted using a InstaGene Matrix (Bio-Rad, USA). MLST was performed using partial sequences of seven house-keeping genes (*adk*, *fumC*, *gyrB*, *icd*, *mdh*, *purA* and *recA*), as previously described. PCR was performed with 20 ng of genomic DNA as template in a 30 μL reaction mixture, using Dr. MAX DNA Polymerase (Doctor protein INC, South Korea) as follows: activation of Taq polymerase at 95 °C for 5 min; 35 cycles at 95 °C for 30 sec, 52 °C for 30 sec, and 72 °C for 1 min; and a final 10 min step at 72 °C. The products obtained after amplification were purified using a multiscreen filter plate (Millipore Corp. USA). Sequencing was performed using a PRISM BigDye Terminator v3.1 Cycle Sequencing Kit. The Mixture was incubated at 95 °C for 5 min, followed by 5 min on ice and then analyzed in an ABI PRISM 3730XL DNA analyzer (Applied Biosystems, USA). Sequence types (ST) were assigned online (http://pubmlst.org/biqsdb?db=pubmlst_ecoli_achtman_seqdef).

### Statistical analysis

Statistical analysis was performed using the SPSS version 12.0 program (SPSS, Chicago, Illinois, USA). Chi-squared test was performed to analyze the pathogenic characteristics and the rate of antimicrobial resistance of *E. coli* from diarrheic pigs and patients.

## Results

### Antimicrobial susceptibility test

**Fig. 1:**
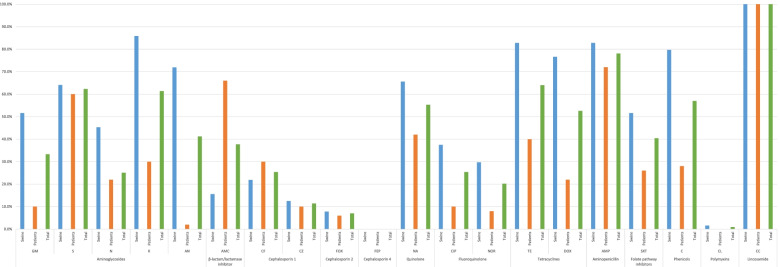
Antimicrobial resistance of *Escherichia coli* from diarrheic pigs and patients. GM: gentamicin; S: streptomycin; N: neomycin; CF: cephalothin; CZ: cefazolin; FEP: cefepime; FOX: cefoxitin; NA: nalidixic acid; CIP: ciprofloxacin; NOR: norfloxacin; AMP: ampicillin; AMC: amoxicillin / clavulanic acid, SXT: trimethoprim / sulfamethoxazole; C: chloramphenicol; CL: colistin; TE: tetracycline. ^*^ Significant difference between origin of isolates (*p* < 0.05). ^**^ Significant difference between origin of isolates (*p* < 0.01)

Figure 1 describes the results of the antimicrobial susceptibility test. The isolates from pigs showed significantly higher resistance to gentamicin (51.6%), kanamycin (85.9%), amikacin (71.9%), nalidixic acid (65.6%), ciprofloxacin (37.5%), norfloxacin (29.7%), tetracycline (82.8%), doxycycline (76.6%), trimethoprim / sulfamethoxazole (51.6%), and chloramphenicol (79.7%) as compared to those from patients (gentamicin: 10.0%, kanamycin: 30.0%, amikacin: 2.0%, nalidixic acid: 42.0%, ciprofloxacin: 10.0%, norfloxacin: 8.0%, tetracycline: 40.0%, doxycycline: 22.0%, trimethoprim / sulfamethoxazole: 26.0%, and chloramphenicol: 28.0%). Isolates from patients showed significantly higher resistance to amoxicillin / clavulanic acid (66.0%) than those from pigs (15.6%). The resistance to cefepime, which is the 4th generation of cephalosporins, was not detected in all isolates.

### Multidrug resistance rates

**Fig. 2:**
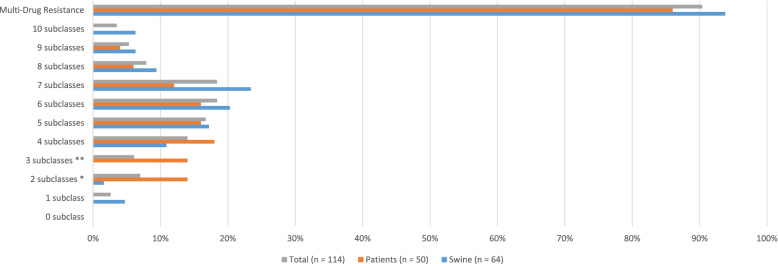
Multidrug resistance of *E. coli* from diarrheic pigs and patients in Korea. Antimicrobial subclasses defined by the Clinical and Laboratory Standards Institute (CLSI) are used. ^*^ Significant difference between origin of isolates (*p* < 0.05). ^**^ Significant difference between origin of isolates (*p* < 0.01)

The results of multidrug resistance analysis are shown in Fig. 2. We found that 23.4% of pig isolates were resistant to seven subclasses (15 isolates), which were the most prevalent, while 18.0% (9 isolates) of isolates of patients showed resistant to four subclasses. Only pig isolates showed resistance to 10 subclasses (4 isolates, 6.3%). In terms of multidrug resistance in those resistant to three or more subclasses of drugs among the 14 subclasses of drugs tested, 93.8% (60 isolates) of pig isolates and 86.0% (43 isolates) of patient isolates showed multidrug resistance.

### Virulence factors

**Fig. 3:**
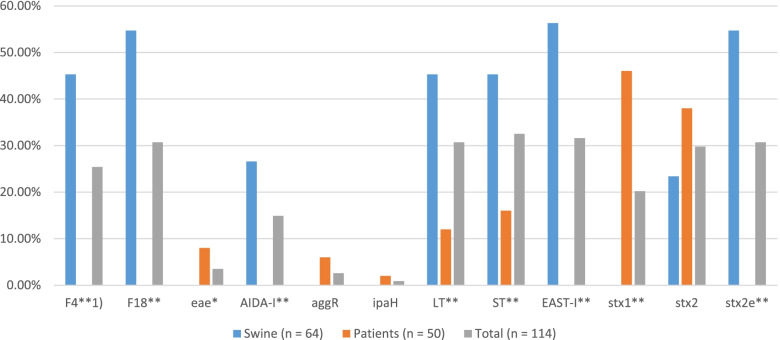
Virulence genes of *E. coli* from diarrheic pigs and patients in Korea. ^*^ Significant difference between origin of isolates (*p* < 0.05). ^**^ Significant difference between origin of isolates (*p* < 0.01)

The prevalence of virulence genes of *E. coli* from diarrheic pigs and patients were compared (Fig. 3). The most prevalent virulence genes in pigs were F18 (35 isolates, 54.7%) and stx2e (35 isolates, 54.7%). While stx1 (23 isolates, 46.0%) was most frequently detected in patients, followed by stx2 (19 isolates, 38.0%). There was no significant difference in the prevalence of stx2 (swine: 23.4%, patients: 38.0%).

### O-serogroups and Virotype

The O-serogroups and virotypes is presented in Table 1. There was no O149 and O139 isolates from patients while most isolates from swine were confirmed in O149 (28 isolates, 43.8%) and O139 (13 isolates, 20.3%). In isolates from patients, O157 (6 isolates, 12.0%) was the most prevalent O-serogroup, and also 2 isolates (3.1%) of swine was confirmed in O157. Interestingly, O157 isolates from swine were ETEC (F4:LT:STb:EAST-I) which is associated with diarrhea, while all isolates from patients were confirmed in STEC (stx1, stx2, and stx1:stx2) which is associated with hemorrhagic colitis.

**Table 1:** Comparison of O-serogroups and virotypes of 114 *Escherichia coli* isolates from diarrheic pigs and patients in Korea

O-serogroup Virotype	No. (%) of pathogenic ***E. coli*** isolates		
	Swine (***n*** = 64)	Patients (***n*** = 50)	Total (***n*** = 114)
**O149**	**28 (43.8%)**	**0 (0.0%)**	28 (24.6%)^**1)^
F4:LT:STb:EAST-I	20	–	20
F18:Stx2e	3	–	3
F18:AIDA-I:Stx2:Stx2e	2	–	2
F18:Stx2:Stx2e	2	–	2
F18:AIDA-I:Stx2e	1	–	1
**O139**	**13 (20.3%)**	**0 (0.0%)**	13 (11.4%)^**^
F18:AIDA-I:Stx2e	13	–	13
**O157**	**2 (3.1%)**	**6 (12.0%)**	**8 (7.0%)**
F4:LT:STb:EAST1	2	–	2
stx1:stx2	–	3	3
stx2	–	2	2
stx1	–	1	1
**O26**	**0 (0.0%)**	**4 (8.0%)**	4 (3.5%)^*^
stx1:stx2	–	2	2
stx1	–	1	1
stx1:eaeA	–	1	1
**O25**	**0 (0.0%)**	**4 (8.0%)**	4 (3.5%)^*^
LT	–	3	3
stx1	–	1	1
**O104**	**0 (0.0%)**	**4 (8.0%)**	4 (3.5%)^*^
stx2:aggR	–	2	2
stx1	–	1	1
stx2	–	1	1
**O159**	**0 (0.0%)**	**4 (8.0%)**	4 (3.5%)^*^
ST	–	3	3
stx1:stx2	–	1	1
**O_UT**^**2)**^	**7 (10.9%)**	**11 (22.0%)**	**18 (15.8%)**
F18:Stx2:Stx2e:EAST-I	4	–	4
AggR	–	3	3
stx1:stx2	–	3	3
stx1	–	2	2
F18:Stx2:Stx2e	2	–	2
stx2	–	1	1
ipaH	–	1	1
F18:Stx2e:EAST-I	1	–	1
−^3)^	–	1	1
**Others**^**4)**^	**14 (21.9%)**	**17 (34.0%)**	**31 (27.2%)**

^1) *^ Significant difference between origin of isolates (*p* < 0.05). ^**^ Significant difference between origin of isolates (*p* < 0.01)

^2)^ O_UT: Untypeable

^3)^ Non-virulence gene detected

^4)^ Other serogroup (number of isolates): O6 (3), O18 (3), O100 (3), O136 (3), O11 (2), O21 (2), O55 (2), O146 (2), O15 (1), O28 (1), O57 (1), O78 (1), O91 (1), O98 (1), O103 (1), O111 (1), O121 (1), O145 (1), O174 (1)

### Multi-locus sequence typing (MLST)

**Fig. 4:**
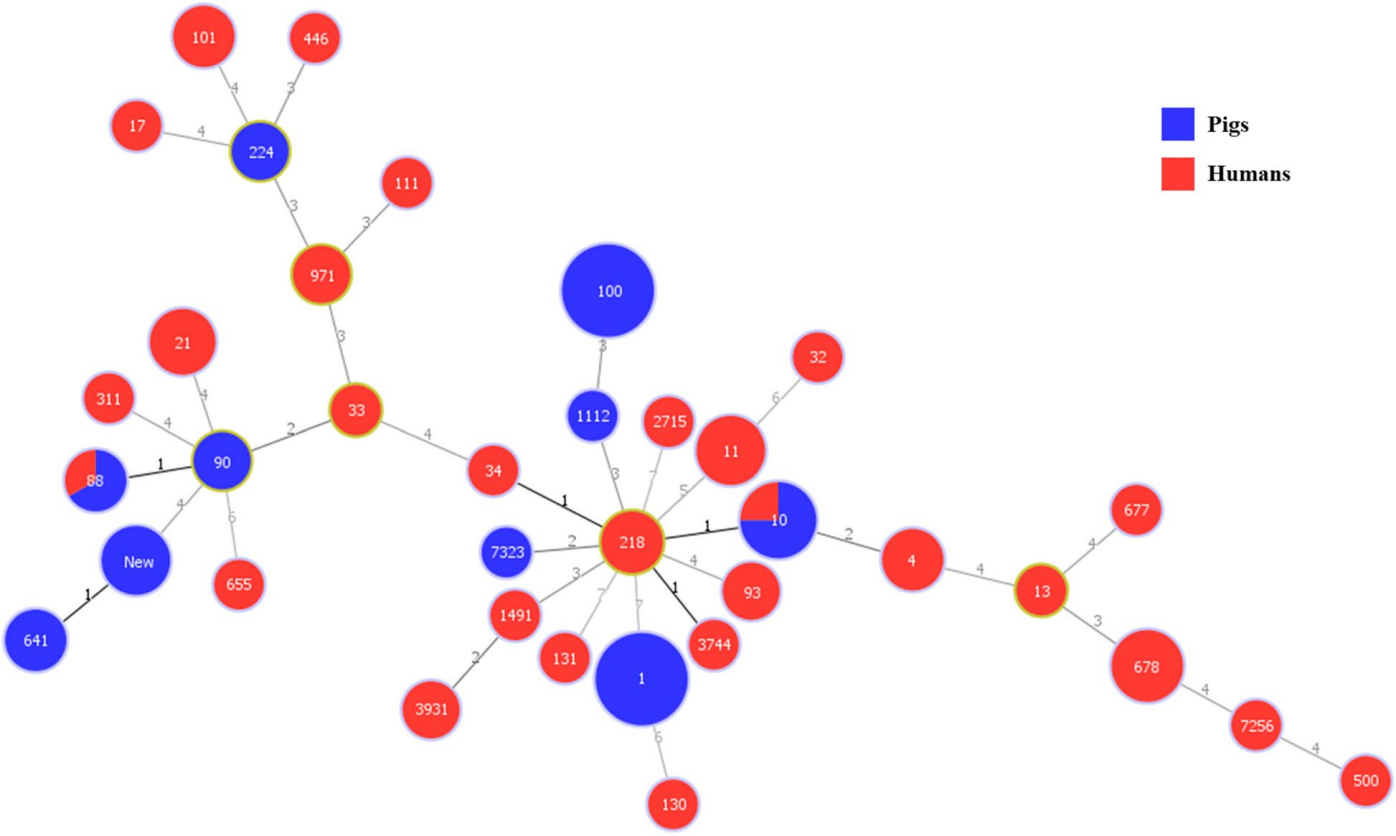
Minimum spanning tree based on sequence type of *E. coli* from pigs and humans

A minimum spanning tree based on MLST data including branch distances is presented in Fig. 4. The divides within each node are equal to the number of isolates belonging to the sequence type it represents. White numbers in the circles indicate the MLST sequence type. Black colored numbers on line indicate the absolute distance between each sequence type. The node sizes vary linearly with the number of isolates of a given sequencing type. The most prevalent ST in swine isolates were ST 1 (21 isolates, 32.8%) and ST 100 (21 isolates, 32.8%). While swine isolates showed only 10 STs, isolates from patients showed 28 STs. The prevalent STs in isolates from patients were ST 678 (6 isolates, 12.0%), ST 21 (4 isolates, 8.0%), and ST 101 (3 isolates, 6.0%). In both swine and patients’ samples, ST 10 (swine: 6 isolates; patients: 2 isolates) and ST 88 (swine: 2 isolates; patients: 1 isolate) were detected simultaneously. Moreover, 5 isolates of swine showed novel STs, ST New. The following STs had 3 absolute distance: ST 34 – ST 218; ST 34 – ST 10; ST 218 – ST 3744; ST 10 – ST 218; ST New – ST 641; ST 88 – ST 90.

## Discussion

It is noted that the overall prevalence of antimicrobial resistance in the isolates from pigs were higher than in those from humans, which was consistent with a previous study [14, 15]. Due to the lack of strict regulations on the use of antimicrobials in Korea [16], using antimicrobials indiscriminately by non-specialists could increase antimicrobial resistance. Isolates from pigs showed significantly higher resistance to aminoglycosides, fluoroquinolone, tetracyclines, nalidixic acid, trimethoprim / sulfamethoxazole, and chloramphenicol than in isolates from patients. The findings that the highest prevalence of resistance occurred among isolates from pigs and that resistance was seen to drug classes approved for use in swine [17, 18] suggest that antimicrobial use in swine may be a factor in the emergence of antimicrobial resistance in *E. coli*. Because these agents are used for treating enteric infections in humans, decreasing resistance to these agents is crucial.

The World Health Organization (WHO) and the World Organization for Animal Health (OIE) have classified fluoroquinolones as “critically important antimicrobial agents” because of their importance in both human and animal medicine [19]. A previous study in Korea reported high resistance to ciprofloxacin (34.5%) in Korean pigs [20]. The present study indicated that resistance of *E. coli* to ciprofloxacin (37.5%) from swine isolates was higher than in countries where the use of antimicrobial agents is restricted (eg. Netherland: 1.0%, Sweden: 0.0%, US: 0.0%) [21, 22]. This is largely due to the massive use of fluoroquinolone in livestock and indiscriminate use by farm workers (quinolone sales: 44,380 kg) in Korea. Moreover, the resistance to ciprofloxacin (swine: 37.5%; patients: 10.0%) and norfloxacin (swine: 29.7%; patients: 8.0%) was higher in swine isolates than in patients’ samples. Since fluoroquinolone resistance can be transferred from pigs to humans [23], this becomes a public health hazard, and it is necessary to establish a strategy to reduce antimicrobial resistance.

In Korea, gentamicin is frequently used for the treatment of colibacillosis [5, 24], whereas it is no longer used in swine farming in advanced countries [25]. This could explain the higher level of resistance in this study (51.6% isolates resistant) compared to published data from other countries (US: 0.0%; Australia: 7.4%) [22, 25]. Moreover, the resistance to some aminoglycoside antimicrobial agents (gentamicin, kanamycin, and amikacin) was significantly higher in isolates from swine than in those from patients. Due to the adverse events associated with aminoglycosides, such as inner ear toxicity (sensorineural hearing loss) and kidney damage (chronic kidney disease), the use of aminoglycosides is limited and administered for severe infections in humans [26]. However, in pigs, aminoglycosides can be used to manage weaning pig scours caused primarily by *E. coli* [26, 27]. The administration of aminoglycosides in pigs has the potential to generate cross-resistance to vitally important human antimicrobials like amikacin, which is a huge concern for human health [25].

In this study, we found high rates of multidrug resistance (swine: 93.8%; patients: 86.0%). Evidently, in our results, values obtained were higher than those obtained in other studies (Pig – Netherland: 34.2% [4], China: 84.2% [28], Thailand: 84.6% [14]; Humans – Netherland: 7.1% [29], China: 15.2% [28], Thailand: 45.7% [14]) In Korea, antimicrobial use in veterinary (33.2 defined daily doses (DDD) per 1000 inhabitants per day) and human medicine (31.7 DDD per 1000 inhabitants per day) is relatively higher as compared to that in other member countries of the Organization for Economic Co-operation and Development (OECD) (21.3 and 23.7 DDD per 1000 inhabitants per day, respectively) [30, 31]. Besides the possible role of increased selective pressure by repeated exposure to therapeutic agents, this is a likely cofactor in the increased frequency of antimicrobial resistance observed among pathogens [32]. The high level of antimicrobial resistance is directly linked to challenges in the treatment of diseases; therefore, it is important to manage antimicrobial resistance.

The most predominant pathotype in Korea was EPEC [33, 34]; however, in this study, the most prevalent pathotype in patients was STEC (27 isolates, 54.0%), followed by ETEC (11 isolates, 22.0%). There was no clear reason why the pathotype of *E. coli* had temporal changes, but it is evident that the predominant pathotype has changed to ETEC and STEC from EPEC. STEC is the third most common zoonotic infection within the Europe [32]. In this study, 28 isolates (43.8%) of swine contained STEC. Because STEC is a zoonotic food- and waterborne pathogen of a serious public health concern and it can cause potentially life-threatening complication, such as hemolytic-uremic syndrome [3, 35], careful attention should be paid to STEC’s pig–human cross-infection.

Fimbriae play an important role in allowing *E. coli* to attach to the intestinal mucosa and epithelial cells [36, 37]. In late 1990s, the most predominant fimbriae in Korean pigs was F6, which then changed to F5 in the mid-2000s [38, 39]. However in this study, there was no fimbrial adhesin in isolates from patients, and the most prevalent virotype of *E. coli* in pigs encoded F4 (29 isolates, 45.3%) and F18 (35 isolates, 54.7%). In Korea, inactivated vaccines targeting F4 and F18 are being used nationwide [40]. The use of these vaccines could cause the antigenic variations and would account for the prevalence of fimbriae or non-fimbrial adhesins, besides F4 and F18, in pigs.

The stx2 gene was detected both in isolates from pigs (15 isolates, 23.4%) and patients (19 isolates, 38.0%). The stx gene was known to be associated with edema disease in swine and hemolytic-uremic syndrome in human [3, 41, 42]. The receptor for stx2 is globotriosyl ceramide which is seen in both swine and humans [43]. Also, the LT and ST gene was detected both in isolates from swine (29 isolates, 45.3%) and patients (11 isolates, 22.0%), which is associated with neonatal or postweaning diarrhea in pigs and in traveler’s diarrhea in humans [44, 45]. There was no common fimbrial adhesin in isolates from both swine and patients. However, several studies reported a high association between non-fimbrial adhesin AIDA-I and F18, which is the most prevalent fimbrial adhesin in the present study [46–48], and AIDA-I was detected in pigs (26.6%), and thus had the potential to cause cross infection between pigs and humans [16]. Furthermore, a recent study indicated that LT also could play a significant role in the enhancement of bacterial adherence [49]. Although no direct transmission could be inferred in this study, the presence of virulence factor, associated with human pathogenicity, in the swine strain gives them potential for cross infection.

There was lower evidence on whether specific O-serogroup could cause diseases because a limited number of O-serogroups have been reported for specific disease [50, 51]. In this study, the most prevalent O-serogroup in swine isolates was O149 (28 isolates, 43.8%), followed by O139 (13 isolates, 20.3%) and O157 (2 isolates, 3.1%). This is in accordance with the results obtained by Kusumoto et al. [52]. Kwon et al. indicated that O157 and O8 were the predominant O-serogroups in Korea from 1995 to 1997 [53]. However, in this study, just two O157 isolates were detected from pigs. The data is suggesting that the predominant serogroup had shifted from O157 to O149 and O139 in Korean swine farms. In contrast to swine isolates, O157 (6 isolates, 12.0%) was the predominant serogroup in patients; O157 is known to be associated with eae, stx1 and/or stx2 gene [54]. Interestingly, swine isolates had no stx2 gene while all isolates from patients encoded stx1 and/or stx2 gene. Because of the low number of O157 strains, it becomes hard to explain the cause of this phenomenon; although further experiments such as whole genome sequencing for address this phenomenon however, we assumed that the relationship of O-serogroup with virotype has been changing over time.

MLST allows determining the phylogenetic relationships among deep lineages, providing a complementary view of the population structure [55]. In this study, there were only 10 STs in swine isolates while patients’ isolates showed more STs (28 STs). Most strains in pigs were ST 1 (21 isolates, 32.8%) and ST 100 (21 isolates, 32.8%), indicating that the cause of enteric colibacillosis in pigs had a similar origin. This is accordance with other studies which state that ST 1 and ST 100 isolates are the predominant ETEC type, and are important pig pathogens in the United States, Canada, Germany, and Thailand (http://mlst.warwick.ac.uk/mlst/dbs/Ecoli). In contrast to swine isolates, each ST of isolates from patients had small portions, which means that each strain from patients had been isolated from a different origin. Several studies reported ST 10 as one of the most common strains in human populations, and ST 10 strains commonly carry certain antimicrobial resistance genes (such as ampC-type beta lactamases and NDM-type carbapenemases) [56, 57]. Also, ST 88 has been previously described in association with c-AmpC production in a French hospital [58]. Interestingly, ST 10 (swine: 6 isolates; patients: 3 isolates) and ST 88 (swine 2 isolates; patients: 1 isolate) were detected simultaneously in swine and patients’ samples. Additionally, we found that isolates from different hosts (swine and patients) were clonally related in minimum spanning tree. In addition, five isolates from swine showing new ST were found that were phylogenetically closed with ST 641. Although, there was a weak relationship between patients’ isolates, the emergence of a pathogenic *E. coli* showing new ST may pose not only a problem in veterinary medicine but also a significant public health threat, and therefore is in need of urgent attention [59]. Similar ST indicates the risk of emergence of zoonotic disease [60] and a risk for cross-infection, and can even cause antimicrobial resistance to be transferred between pigs and humans in Korea.

## Conclusions

In this study, we analyzed antimicrobial resistance, virulence genes, O-serogroups, and MLST of *E. coli* from isolates of pigs and patients suffering from diarrhea. Both isolates from swine and patients had the stx2 gene, which could cause severe disease, such as edema disease (swine) and hemorrhagic colitis (human). Isolates from swine showed significantly higher antimicrobial resistance than those from humans, especially in fluoroquinolone and aminoglycosides. Through these results, we could assume that the pig could act as a reservoir in human infection. Also, pigs are a reservoir for bacteria with high resistance rate to antimicrobial agents, especially compared to countries where the use of antimicrobials are limited. These results provide important data that can be used to support the development of vaccines to implement strategies for the control and prevention of antimicrobial resistance.

## Supplementary Information

**Additional file 1.**

## Data Availability

The datasets generated and/or analyzed during the current study are available from the corresponding author on reasonable request.
